# New 5-Aryl-1,3,4-Thiadiazole-Based Anticancer Agents: Design, Synthesis, In Vitro Biological Evaluation and In Vivo Radioactive Tracing Studies

**DOI:** 10.3390/ph15121476

**Published:** 2022-11-27

**Authors:** Rana M. El-Masry, Basma M. Essa, Adli A. Selim, Soad Z. El-Emam, Khaled O. Mohamed, Tamer M. Sakr, Hanan H. Kadry, Azza T. Taher, Sahar M. Abou-Seri

**Affiliations:** 1 Organic Chemistry Department, Faculty of Pharmacy, October University for Modern Sciences and Arts (MSA), October 6 City 12451, Egypt; 2 Radioactive Isotopes and Generator Department, Hot Labs Center, Egyptian Atomic Energy Authority, P.O. Box 13759, Cairo 13759, Egypt; 3 Labeled Compounds Department, Hot Labs Center, Egyptian Atomic Energy Authority, P.O. Box 13759, Cairo 13759, Egypt; 4 Department of Pharmacology and Toxicology, Faculty of Pharmacy, October 6 University (O6U), October 6 City 12585, Egypt; 5 Department of Pharmaceutical Organic Chemistry, Faculty of Pharmacy, Cairo University, Cairo 11565, Egypt; 6 Department of Organic Pharmaceutical Chemistry, Faculty of Pharmacy, October 6 University (O6U), October 6 City 12585, Egypt; 7 Department of Pharmaceutical Chemistry, Faculty of Pharmacy, Cairo University, Cairo 11565, Egypt

**Keywords:** 1,3,4-thiadiazole, anticancer activity, structure-activity relationship, radiolabeling, in vivo pharmacokinetics

## Abstract

A new series of 5-(4-chlorophenyl)-1,3,4-thiadiazole-based compounds featuring pyridinium (**3**), substituted piperazines (**4a–g**), benzyl piperidine (**4i**), and aryl aminothiazoles (**5a–e**) heterocycles were synthesized. Evaluation of the cytotoxicity potential of the new compounds against MCF-7 and HepG2 cancer cell lines indicated that compounds **4e** and **4i** displayed the highest activity toward the tested cancer cells. A selectivity study demonstrated the high selective cytotoxicity of **4e** and **4i** towards cancerous cells over normal mammalian Vero cells. Cell cycle analysis revealed that treatment with either compound **4e** or **4i** induced cell cycle arrest at the S and G2/M phases in HepG2 and MCF-7 cells, respectively. Moreover, the significant increase in the Bax/Bcl-2 ratio and caspase 9 levels in HepG2 and MCF-7 cells treated with either **4e** or **4i** indicated that their cytotoxic effect is attributed to the ability to induce apoptotic cell death. Finally, an in vivo radioactive tracing study of compound **4i** proved its targeting ability to sarcoma cells in a tumor-bearing mice model.

## 1. Introduction

Cancer is a genetic disease, triggered by changes in genes controlling cell function, growth, and proliferation. Cancer’s rising prominence as a leading cause of death partly reflects marked declines in mortality rates of stroke and coronary heart disease, relative to cancer, in many countries. The most recent annual world report by the IARC (International Agency for Research on Cancer) states that in 2020 there were 19.3 million new cases and 10.0 million cancer-related deaths [1]. That year, female breast cancer overtook lung cancer as the most diagnosed cancer type, accounting for 2.3 million new cases (11.7%). Liver cancer is the second leading cause of cancer death, with approximately 830 thousand deaths (8.3%) [1]. Chemotherapy as a main strategy for treatment is highly toxic to normal cells with severe side effects even at therapeutic doses [2]. Therefore, the synthesis of new selective anti-cancer agents with high safety margins is a rich and interesting topic for researchers.

**Figure 1:**
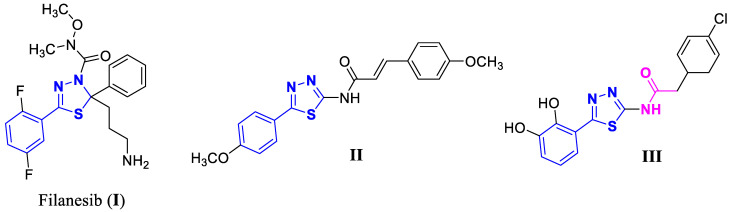
Structures of 1,3,4-thiadiazole derivatives (**I**–**III**) with anticancer activity.

Heterocycle-based compounds are valuable scaffolds in anticancer drug discovery as they can easily target various metabolic pathways and cellular processes in cancer pathology [3]. One of the most advantageous heterocycles is the 1,3,4-thiadiazole, a five-membered ring used as a pharmacophore with versatile biological activities and applications due to its favorable metabolic profile and ability to form hydrogen bonds. Moreover, the sulfur atom of 1,3,4-thiadiazole imparts improved lipid solubility [4]. The mesoionic nature of this class of compounds allows it to easily cross cellular membranes, showing good tissue permeability [3,4]. Thus, the 1,3,4-thiadiazole ring emerged as a prominent pharmacophore in the synthesis of anticancer agents. Filanesib (**I**) is a kinesin spindle protein (KSP) inhibitor used in clinical trials for patients with multiple myeloma, advanced/refractory myeloid leukemia, and advanced solid tumors [5]. The reported 1,3,4-thiadiazole derivative (**II)** displayed potent growth inhibitory activity against breast cancer MCF-7 cells (IC_50_ = 0.28 µg/mL) through cell cycle arrest at the G2/M phase [6]. Additionally, the aryl acetamide 1,3,4-thiadiazole derivative (**III)** inhibited the survival of acute promyelocytic leukemia HL-60 cells (IC_50_ = 9.6 µM) and down-regulated the MMP2 and VEGFA expression levels in the treated cells compared to the control cell samples [7] (Figure 1).

**Figure 2:**
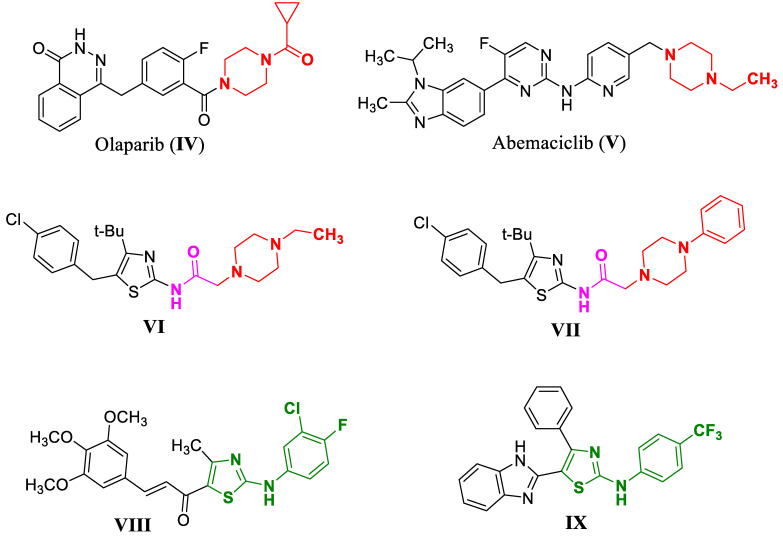
Structures of piperazine-bearing compounds and (**IV**–**VII**) aryl aminothiazole-bearing compounds (**VIII**,**IX)** with anticancer activity.

The piperazine ring is a key component in a large number of reported cytotoxic agents because of its ability to improve pharmacological and pharmacokinetic profiles of drug candidates through tuning of target interactions as well as enhancing aqueous solubility and bioavailability [8]. Piperazine derivatives demonstrated potent antiproliferative activities against numerous cancer cell lines, including colon, prostate, breast, lung, and leukemia, and showed effective suppression of experimental tumor models by multiple mechanisms including the inhibition of microtubule synthesis, cell-cycle progression, angiogenesis, and protein kinases [9]. Olaparib (**IV**) and Abemaciclib (**V**) are piperazine-based drugs approved by the FDA as targeted therapies for breast cancer [10]. Moreover, the incorporation of an ethyl piperazine moiety in compound **VI** remarkably increased the antitumor activity against breast cancer MCF-7 cells (IC_50_ = 12.5 µM) compared to the lead compound, while introducing phenyl piperazine moiety in **VII** caused a significant increase in cytotoxicity against lung A549 (IC_50_ = 0.2 µM) and cervical HeLa (IC_50_ = 4.2 µM) cancer cells [11] (Figure 2). 

**Figure 3:**
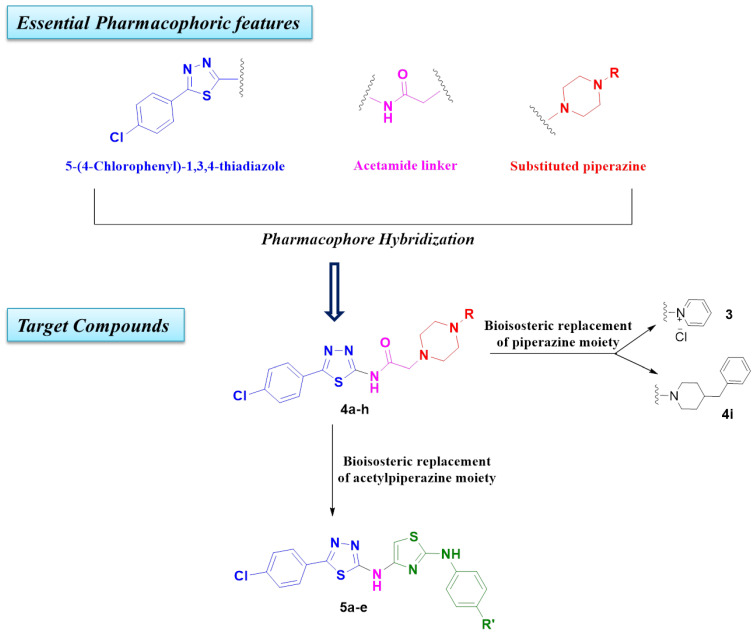
Design of target compounds **3, 4a**–**i** and **5a**–**e**.

Based on the aforementioned information, we predicted that the integration of the unique advantages of thiadiazole as an anticancer pharmacophore with piperazine may improve the anticancer potential of the new-chemical entities. Accordingly, we adopted the pharmacophoric hybridization strategy by attaching the 5-(4-chlorophenyl)-2-amino-1,3,4-thiadiazole scaffold with substituted piperazines through an acetamide linker, hoping that the new hybrids **4a–h** may have enhanced antitumor activity. The choice of the 5-(4-chlorophenyl)-1,3,4-thiadiazole scaffold as basic pharmacophore in the design of the new hybrids was based on the reported fact that substitution on the 5-phenyl with the electron-withdrawing chlorine atom boosted the cytotoxic activity of the thiadiazole derivatives [4,12]. For comparative reasons, we decided to study the effect of the replacement of the substituted piperazines in **4a–h** with the pyridinium ring in **3** and benzyl piperidine in **4i** (Figure 3). 

On the other hand, aryl aminothiazole is another important class of compounds with notable antiproliferative activity [13]. The aryl aminothiazole chalcone **VIII** effectively restrained the growth of human gastric cancer BGC-823 and human lung carcinoma NCI-H, as compared to the clinical drug Cisplatin [14], while benzimidazole aminothiazole **IX** was identified as a cytotoxic agent with low micromolar potency toward human lung cancer [13] (Figure 2). Hence, the second approach adopted for this study was the bioisosteric replacement of the substituted acetylpiperazine fragment in **4a–h** with an aryl aminothiazole moiety in compounds **5a–e** (Figure 3). 

The synthesized compounds **3**, **4a–i**, and **5a–e** were evaluated for their cytotoxic activity in vitro against MCF-7 and HepG2 cancer cell lines. In addition, a flow cytometric analysis of apoptotic cell death and cell cycle phase distribution analysis, and determination of the Bax/bcl2 ratio, caspase 9, and VEGF levels were performed for cancer cells treated with the most potent compounds, **4e** and **4i**. Finally, the pharmacokinetic behavior of the most active compound, **4i,** was studied with the aid of a radiolabeling technique to evaluate its targeting ability to a solid tumor in a sarcoma-bearing mouse model.

## 2. Results and Discussion

### 2.1. Chemistry

**Scheme 1:**
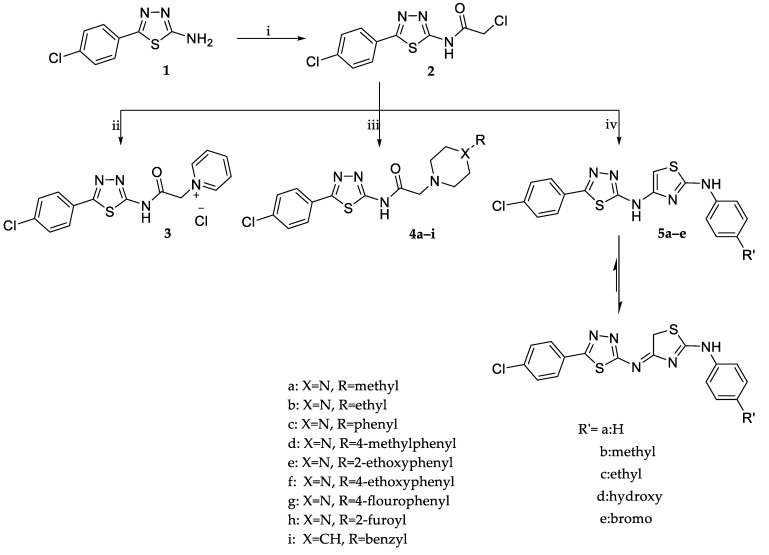
The synthetic approaches adopted for the synthesis of target compounds. Reagents and conditions: (i) Chloroacetyl chloride, anhydrous sodium acetate, dry acetone, r.t. 1 h, yield= 87%; (ii) Dry pyridine, reflux 6 h, yield= 81%; (iii) Secondary amines, TEA, dry benzene, reflux 16–20 h, yield= 61–89%; (iv) Thiourea deivatives, anhydrous sodium acetate, absolute ethanol, reflux 4–5 h, yield= 50–56%.

The synthetic approaches adopted for the synthesis of target compounds are illustrated in Scheme 1. 5-(4-Chlorophenyl)-1,3,4-thiadiazol-2-amine **1 was** reacted with chloroacetyl chloride in the presence of anhydrous sodium acetate to yield the key intermediate 2-chloro-*N*-(5-(4-chlorophenyl)-1,3,4-thiadiazol-2-yl)acetamide (**2**)**.** The latter was subjected to a nucleophilic substitution reaction by heating under reflux with dry pyridine or substituted piperazines/benzyl piperidine in dry benzene containing a catalytic amount of triethyl amine to yield compounds **3** and **4a–i,** respectively. Spectral and elemental analysis data were in full agreement with the postulated structures of compounds **3** and **4a–i**. For example, the ^1^H NMR spectrum of the 4-ethoxyphenyl piperazine derivative (**4f**) revealed the presence of two broad singlet signals at δ 2.69 and 3.17 ppm along with a singlet signal at δ 3.46 ppm assigned for the piperazine and the acetamide CH_2_ protons, respectively, confirming the success of the substitution reaction. Moreover, the characteristic triplet-quartet signals of the 4-ethoxy group were detected at δ 1.20 and 3.03 ppm. The ^13^C NMR spectrum of compound **4f** showed five signals at the aliphatic region: at δ 8.93 and 45.92 ppm, attributed to the ethoxy group carbons, and at δ 48.39 and 52.67 ppm due to the piperazine carbons. While the signals of the acetamide linker carbons resonated at δ 60.26 ppm for the CH_2_ carbon and δ 169.18 ppm for the carbonyl carbon.

Alternatively, the 2-chloroacetamide intermediate **2** was reacted with substituted thiourea derivatives. The reaction involves the base-catalyzed *s*-alkylation via nucleophilic substitution of the chlorine atom in **2** followed by in situ cyclodehydration to yield the aryl aminothiazoles **5a–e.** The structures of compounds **5a–e** were confirmed by spectroscopic methods, IR, ^1^H NMR, and ^13^C NMR. The IR spectrum of compound **5b** revealed the disappearance of the C = O absorption band at 1706 cm^−1^ characteristic of the amide function in intermediate **2**, which confirmed the cyclocondensation reaction. Compounds **5a–e** have two tautomeric forms, the aminothiazole tautomer and the iminodihydrothiazole one. The dominance of the iminodihydrothiazole form was evident from the NMR spectral data. The ^1^H NMR spectrum of **5b** showed two singlet signals integrated for three and two protons at δ 1.76 and 2.18 ppm assigned to the *p*-tolyl CH_3_ and the dihydrothiazole methylene (CH_2_) protons, respectively, while the signals due to the methyl and methylene carbons appeared at δ 20.92 and 23.91 ppm in its ^13^C NMR spectrum. Furthermore, the ^1^H NMR spectrum revealed the appearance of two new doublet signals at δ 7.08 and 7.90 ppm corresponding to the AB system of the *p*-tolyl ring which was confirmed by the increase in the number of signals of the aromatic carbons in the ^13^C NMR spectrum.

### 2.2. Biological Evaluation

#### 2.2.1. In Vitro Cytotoxic Activity

The in vitro cytotoxicity of the synthesized 1,3,4-thiadiazole derivatives against breast adenocarcinoma cells (MCF-7) and human hepatocellular carcinoma (HepG2) cell lines were evaluated by the MTT assay, using 5-Fluorouracil (5-FU) as a positive control. The results were expressed by a median inhibitory concentration (IC_50_ in µg/mL) as presented in Table 1. The tested compounds displayed a wide range of anticancer activity with IC_50_= 2.34–91.00 µg/mL against MCF-7 and IC_50_= 3.13 to 44.87 µg/mL against HepG2. Compounds **4i**, **4h**, **5e**, **4e,** and **5c** (IC_50_= 2.32, 3.21, 3.77, 5.36, and 5.72 µg/mL, respectively) were the most potent against MCF-7, whereas compounds **5d** and **3** (IC_50_= 6.12 and 7.56 µg/mL) showed comparable activity to 5-FU (IC_50_= 6.80 µg/mL). For the HepG2 cell line, compounds **4e**, **4b,** and **4i** (IC_50_= 3.13–6.51 µg/mL) showed stronger cytotoxicity than 5-FU (IC_50_= 8.40 µg/mL), while compounds **4h** and **5b** (IC_50_= 8.35 and 8.81 µg/mL) were equipotent to 5-FU. 

The structure-activity relationship was extracted by comparing the IC_50_ values of the synthesized compounds presented in Table 1. For the MCF-7 cell line, expanding the 5-(4-chlorophenyl)-1,3,4-thiadiazole moiety with a pyridinium ring through an acetamide linker, derivative **3,** showed good activity similar to 5-FU (IC_50_= 7.56, 6.80 µg/mL). 

Regarding the piperazine derivatives **4a–h,** it was noticed that the antitumor potency was affected by the substituents on N_4_ of piperazine. The 4-methyl piperazine derivative **4a** resulted in fair activity (IC_50_= 51.56 µg/mL). A two-fold increase in potency was obtained upon elongation of the methyl group in **4a** into ethyl in **4b** (IC_50_= 25.21 µg/mL). Replacement of the **4a** methyl group by a phenyl ring resulted in the equipotent compound **4c** (IC_50_= 49.40 µg/mL). Grafting a *p*-methyl group to the phenyl ring of **4c** afforded compound **4d** with more than a 2-fold increase in activity. Further improvement in activity was observed upon substituting the phenyl ring of compound **4c** at para-position with the electron-donating ethoxy group **4f** (IC_50_= 18.39 µg/mL) or electron-withdrawing fluoro atom **4g** (IC_50_= 10.10 µg/mL). Interestingly, shifting the position of the ethoxy group from the *para*-position in **4f** to the *ortho*-position in **4e** caused about four times increase in activity (IC_50_= 5.36 µg/mL). Furthermore, the bioisosteric replacement of the terminal phenyl ring on the piperazine in **4c** with a furoyl moiety in **4h** highly augmented the antiproliferative activity (IC_50_= 3.21 µg/mL). Remarkably, substituting the phenyl piperazine moiety in **4c** with benzyl piperidine moiety afforded the most potent antitumor compound in this study, **4i** (IC_50_= 2.32 µg/mL).

The derivatives bearing the arylamino thiazole scaffold, **5a–e**, exhibited potent to moderate activity against the MCF-7 cell line (IC_50_= 3.77–24.79 µg/mL); the unsubstituted phenyl derivative **5a** (R’ = H) showed moderate activity (IC_50_= 24.79 µg/mL). *Para*-substitution on the phenyl ring in **5a** with either hydrophobic **5b** (R’= methyl), **5c** (R’ = ethyl), or hydrophilic **5d** (R = hydroxyl) electron donating groups resulted in a 2- to 4-fold improvement in activity (IC_50_= 5.72–12.60 µg/mL). Meanwhile substitution with a lipophilic electron withdrawing bromine atom resulted in one of the most potent compounds against MCF-7, **5e** (IC_50_= 3.77 µg/mL).

Concerning the HepG2 cell line, it was revealed that most of the tested compounds showed potent to moderate activity compared to 5-FU (IC_50_= 8.40 µg/mL), with IC_50_ values ranging from 3.13 to 44.87 µg/mL. In analogy to MCF-7, increasing the size of the aliphatic substituent on N_4_ of piperazine from methyl **4a** (IC_50_= 26.30 µg/mL) to ethyl **4b** (IC_50_= 3.55 µg/mL) and shifting the *para*-ethoxy substituent in **4f** (IC_50_= 38.10 µg/mL) to the *ortho*-position in **4e** (IC_50_= 3.13 µg/mL) produced the most effective compounds against the HepG2 cell line with a 7- and 12-fold increase in efficacy, respectively. Furthermore, the replacement of the phenyl ring in compound **4c** (IC_50_= 27.94 µg/mL) with furoyl moiety, **4h** (IC_50_= 8.35 µg/mL), or substituting the phenyl piperazine **4c** with the benzyl piperidine scaffold **4i** (IC_50_= 6.51 µg/mL) led to compounds with a high antitumor activity that is comparable to 5-FU. 

In contrast to the MCF-7 cell line, substitution on the phenyl ring of the arylamino thiazole derivative **5a** (IC_50_= 26.12 µg/mL) with hydrophobic (**5b:** R’ = CH_3_; IC_50_= 8.81 and **5c** R’ = C_2_H_5_; IC_50_= 11.25 µg/mL) or hydrophilic (**5d:** R’ = OH; IC_50_= 14.12 µg/mL) electron donating groups enhanced the antiproliferative activity, while substitution with an electron-withdrawing bromine atom formed compound **5e** with similar cytotoxicity (IC_50_= 25.77 µg/mL). 

In summary, the antitumor activity of the novel 5-(4-chlorophenyl)-1,3,4-thiadiazoles against MCF-7 and HepG2 cell was enhanced by expansion with a piperazine or piperidine ring featuring a more lipophilic *o*-ethoxyphenyl, **4e,** or benzyl moiety, **4i,** through an acetamide linker. In addition, the bioisosteric replacement of the *o*-ethoxyphenyl in **4e** with a furoyl moiety in **4h** improved the activity against MCF-7 cells. 

The results showed that compounds **4e** and **4i** highly restrained the growth of both MCF-7 and HepG2 cell lines and were more potent than 5-FU. In order to determine their selectivity, and hence their safety, compounds **4e** and **4i** and their antiproliferative activity was tested against normal mammalian Vero cells and selectivity indices (SI) were calculated and illustrated in Table 2. Compounds **4e** and **4i** displayed weak or no cytotoxic effect on normal cells (IC_50_ = 154.30 and 85.29 µg/mL, respectively) with high selective cytotoxicity for MCF-7 (SI = 28.79 and 36.70) and HepG2 (SI = 49.20 and 13.10) cells over normal Vero cells, indicating that these compounds are potent and selective antitumor agents.

#### 2.2.2. Evaluation of Cellular Death Pattern Induced by **4e** and **4i**

Morphologically, there are three different modes of cell death: apoptosis, autophagy, and necrosis (types I, II, and III, respectively) [15]. Apoptosis and necrosis are commonly elicited when cancer cells are exposed to cytotoxic agents [16], therefore, we evaluated the pattern of cancer cell death induced after treatment with the most potent compounds **4e** and **4i** at their IC_50_ concentrations for 48 h by morphologic evaluation using AO/EB fluorescent stain and flow cytometric analysis.

##### Morphological Evaluation of Apoptotic and Necrotic Cells Using AO/EB Stain

**Figure 4:**
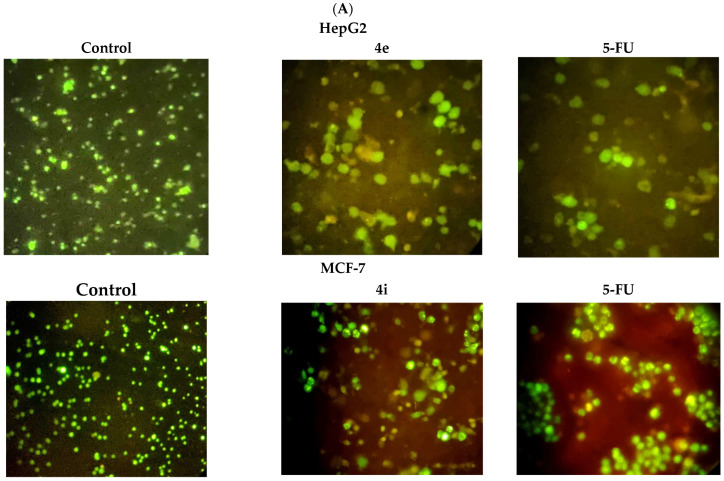

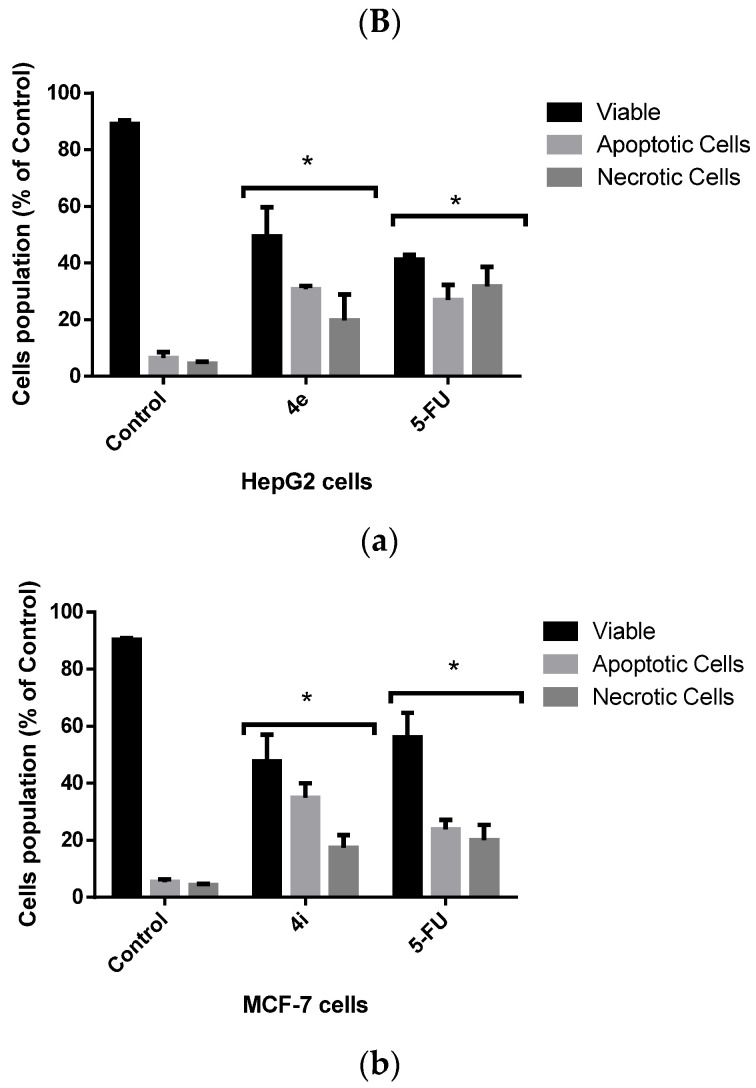
(**A**) HepG2 and MCF-7 cells were stained with the AO/EB fluorescent dye to distinguish between different cell populations: live (green), apoptotic (yellow to light orange), and necrotic cells (dark orange to red) and viewed under a fluorescence microscope. (**B**) The percentage of distinct cell populations in HepG2 (**a**) and MCF-7 cells (**b**). * *p* < 0.05.

Treatment with either compound **4e** or **4i** significantly enhanced apoptosis and necrosis in HepG2 and MCF-7 cells, respectively, when compared with untreated control cells. On the other hand, there was no significant difference between the **4e** and **4i** compounds and 5-FU, the positive control (Figure 4). This indicates that **4e** and **4i** are as effective in inducing cytotoxic effects against HepG2 and MCF-7 cells, respectively, as the commercial drug 5-FU.

##### Flow Cytometric Analysis of Apoptotic Cell Death

**Figure 5:**
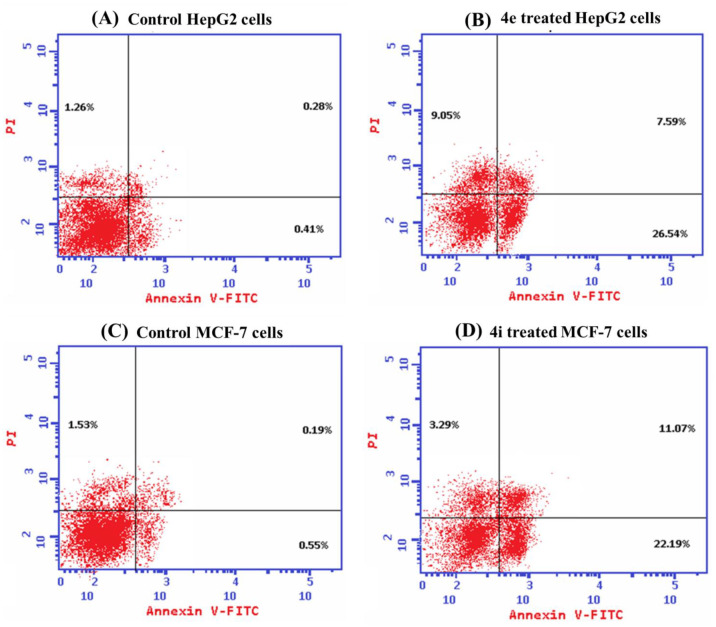

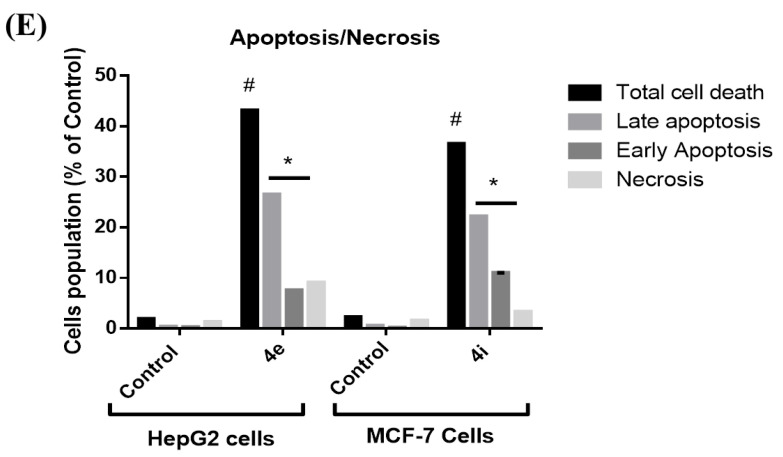
An annexin-V/PI stain for studying apoptosis by flow cytometric analysis. The representative histogram of different cell populations; viable, early apoptotic, late apoptotic, and necrotic cells for (**A**) control HepG2 cells; (**B**) **4e** treated HepG2 cells; (**C**) control MCF-7 cells; and (**D**) **4i** treated MCF-7 cells. (**E**) A minimum of 10,000 cells per sample were used to calculate the percentage of various cell populations used to determine cell death patterns. Within each experiment, data are presented as the mean standard deviation for triplicates. The one-way ANOVA was used for statistical analysis. * *p* < 0.05 vs. Necrosis; # *p* < 0.05 vs. Control.

Propidium iodide (PI) and annexin-V-FITC were used in a flow cytometric investigation on HepG2 and MCF-7 cells to better understand the manner of cell death caused by **4e** and **4i**, respectively. Both **4e** and **4i** compounds induced significant total cell death when compared with untreated cells; additionally, the main mode of cell death was apoptosis as the percentage of the total apoptotic cell populations was significantly higher than that of the necrotic cells (Figure 5). Apoptosis evasion is one of the hallmarks of cancer progression because the loss of apoptosis control prolongs cancer cell survival and allows for the accumulation of mutations that can promote angiogenesis and cell proliferation, disrupt differentiation, and increase invasiveness throughout tumor progression, thus one of the key purposes of apoptosis is to prevent cancer [17,18].

#### 2.2.3. Cell Cycle Analysis

**Figure 6:**
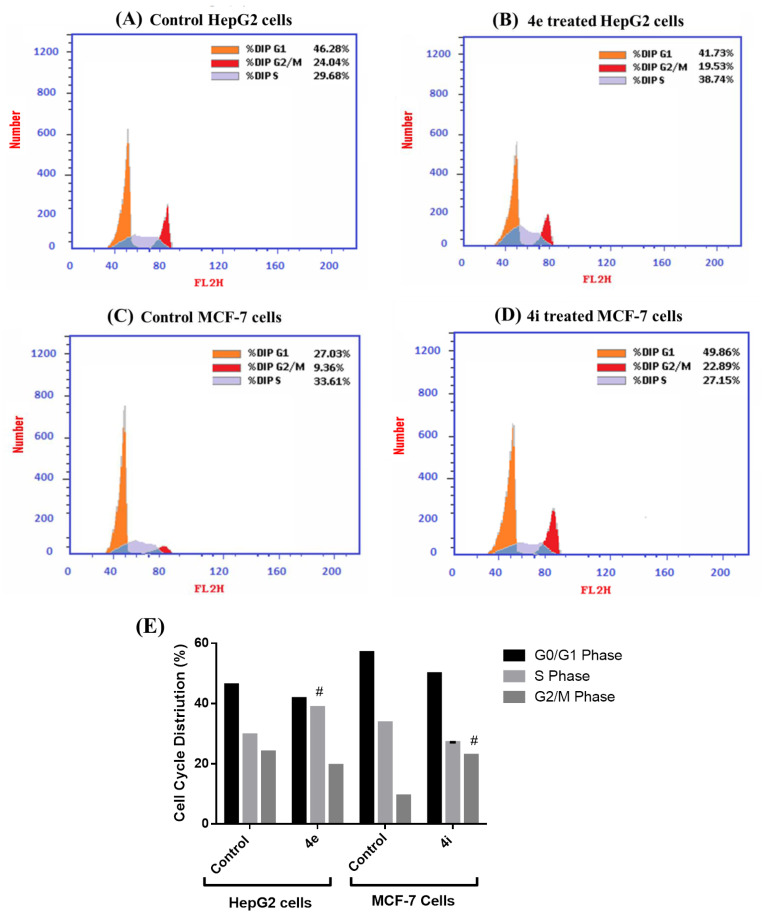
The cell cycle analysis using flow cytometry in HepG2 and MCF-7 cells. The representative histogram of the G0/G1, S, and G2/M phases of the gated cells for (**A**) control HepG2 cells; (**B**) **4e** treated HepG2 cells; (**C**) control MCF-7 cells; and (**D**) **4i** treated MCF-7 cells. (**E**) The percentage of cell cycle distribution: each sample’s cell distribution was quantitatively examined using at least 10,000 cells per sample. Within a single experiment, data are presented as the mean standard deviation for triplicates. The one-way ANOVA was used for statistical analysis. # *p* < 0.05 compared to Control.

The cell cycle is made up of several intricately linked processes that enable the cell to develop and multiply. In cancer, the genetic and epigenetic abnormalities in the cell cycle-related genes result in an unchecked proliferation of mutated cells and alteration of cell cycle progression [19]. For further investigation of the inhibitory effects of **4e** and **4i** compounds on the proliferation of HepG2 and MCF-7 cells, respectively, we examined the cell cycle distribution and DNA content after 48 h of the treatment. Our results showed that the **4e** compound significantly arrested HepG2 cells in the S phase, which is the most critical point for cellular proliferation, confirming the antiproliferative effect of **4e** against HepG2 cells [20]. On the other hand, the **4i** compound induced a significant cell growth arrest for MCF-7 cells at the G2/M phase when compared to control cells (*p* < 0.05) as presented in Figure 6. Consequently, the arrest in the G2/M phase mediates apoptosis via a mitochondrial apoptotic pathway [20]. Our results indicate that both compounds, **4e** and **4i**, induce cell cycle arrest as a result of DNA damage, thus, they are promising cytotoxic agents against cancer cells.

#### 2.2.4. Effects of **4e** and **4i** Compounds on the Bax/Bcl2 Ratio, Caspase 9, and VEGF Levels in Cancer Cells

**Figure 7:**
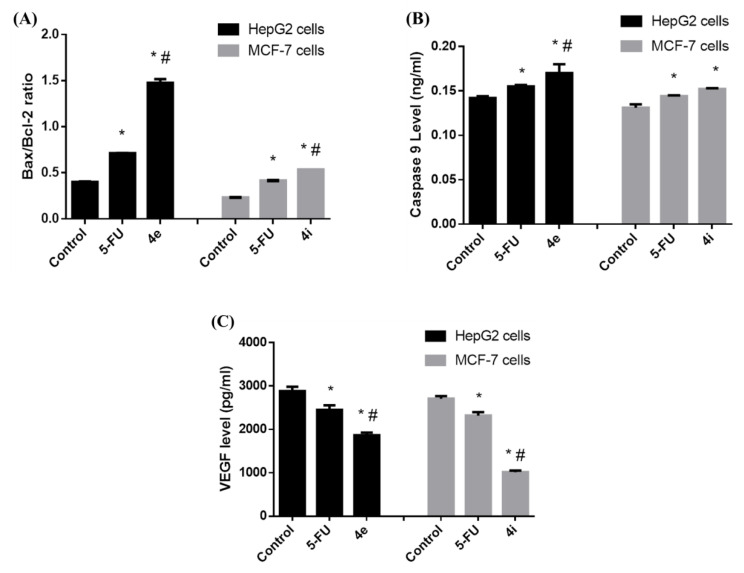
The effect of **4e** and **4i** compounds on the (**A**) Bax/Bcl-2 ratio, (**B**) caspase 9 level, and (**C**) VEGF level. Data are expressed as the mean ± standard deviation for triplicates within an individual experiment. Statistical significance was performed using one-way ANOVA. * *p* < 0.05 vs. Control, # *p* < 0.05 vs. 5-FU.

The overexpression of antiapoptotic proteins, such as Bcl-2, and the underexpression of proapoptotic proteins, such as Bax, are two common ways that the apoptotic pathway is suppressed in cancer [21]. Additionally, this contributes significantly to their resistance to chemotherapy. Therefore, a rise in Bax expression and a decrease in Bcl-2 can trigger the apoptotic process and eradicate cancer cells [22]. Our results showed that the **4e** and **4i** compounds induced a significant increase in the Bax/Bcl-2 ratio in HepG2 and MCF-7 cells, respectively, as compared to control and 5-FU treated cells. This in turn activates the intrinsic pathway of apoptosis; thus, we next examined their effect on caspase 9 levels. Caspases (cysteine aspartyl-specific proteases), a class of cysteine proteins that cleave target proteins, play a role in executing apoptosis [23]. Caspase 9 is well known for its function as an intrinsic apoptosis initiator and its level was significantly enhanced by both **4e** and **4i** compounds. Moreover, we evaluated the effect of the two compounds on the vascular endothelial growth factor VEGF expression. VEGF is one of the pro-angiogenic proteins; upon binding to VEGF receptors in the endothelial cells, it activates phosphorylation, causing increased angiogenesis and resulting in abnormal vasculature in the tumor tissue [24,25]. The **4e** and **4i** compounds significantly suppressed the levels of VEGF in HepG2 and MCF-7 cells, respectively, when compared to control and 5-FU treated cells (Figure 7). 

In summary, the increase in the Bax/Bcl2 ratio and caspase 9 levels and the decrease in VEGF levels revealed that compounds **4e** and **4i** enhance cell death via apoptosis and induce suppression of angiogenesis by reducing VEGF.

#### 2.2.5. Radiosynthesis and In Vivo Biodistribution of Radioiodinated-**4i**

The radiopharmaceutical chemistry field represents an intensive approach to studying the ADME parameters of newly synthesized molecules [26]. Radioactive iodine is very efficient for the radioiodination of organic compounds to act as a screening probe of their in vivo biodistribution pattern as radioiodine is effectively compatible with a wide scope of organic compounds, in addition to its easy physical radio-imaging screening [27].

Since compound **4i** demonstrated the highest cytotoxicity among all the tested compounds. It was selected for radiolabeling and biodistribution studies. 

##### Radiosynthesis of Radioiodinated-**4i**

**Figure 8:**
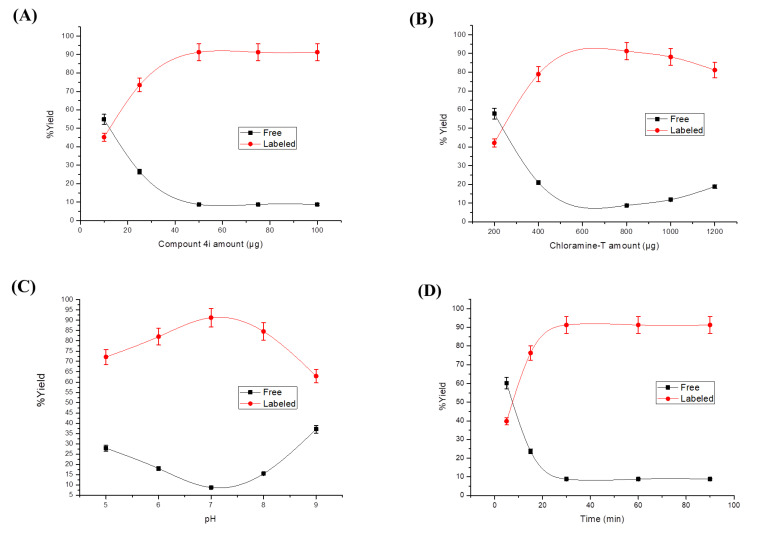
The variation of radiosynthesis yield of the radioiodinated-**4i** compound in relation to (**A**) compound **4i** amount, (**B**) chloramine-T amount, (**C**) pH, and (**D**) reaction time.

Radioiodinated-**4i** was successfully radiosynthesized, showing a high radiosynthesis yield of 91.24 ± 1.49% that was attained using 800 μg chloramine-T, pH 7, 50 μg of compound **4i,** and 30 min of reaction time as presented in Figure 8A–D.

##### Radioactive In Vivo Tracing Studies of Radioiodinated-**4i** in a Sarcoma-Bearing Mice Model

**Figure 9:**
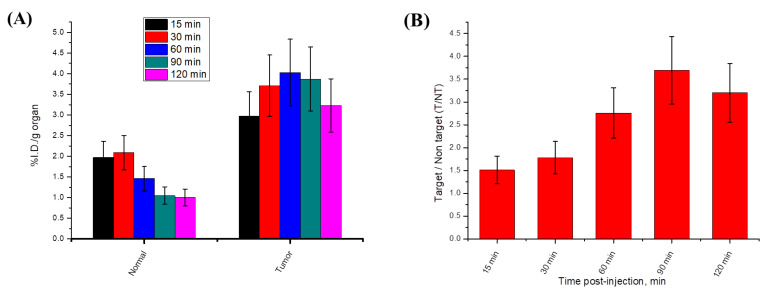
(**A**) Percent injected dose per gram organ of normal and tumor muscles and (**B**) target/non-target ratios of radioiodinated-**4i** in sarcoma-bearing mice model.

**Figure 10:**
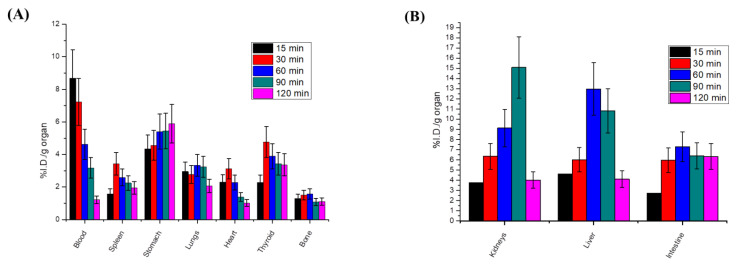
Percent injected dose per gram organ of the radioiodinated-**4i** compound in sarcoma-bearing mice model in (**A**) non-target organs and (**B**) excretory organs.

We studied the cancer-targeting ability of radioiodinated-**4i** in a sarcoma-bearing mice model. As illustrated in Figure 9A, the solid tumor showed a higher %ID/g in all time intervals compared to normal muscle. In addition, the target/non-target ratio of radioiodinated-**4i** was greater than 1 at all-time intervals and reached its maximum value at 1.5 h post-injection (T/NT = 3.69) Figure 9B. These results indicated that radioiodinated-**4i** was able to efficiently target cancer tissue. Biodistribution studies clarified that there was no significant accumulation of the radioiodinated-**4i** compound in any of the non-target organs and revealed a normal blood clearance pattern Figure 10A. Moreover, the highest %ID/g of radioiodinated-**4i** was detected in the excretory organs, i.e., the kidneys, liver, and intestine, Figure 10B, which means that radioiodinated-**4i** was mainly excreted via renal and hepatobiliary routes.

## 3. Materials and Methods

### 3.1. Chemistry

#### 3.1.1. General

A Stuart melting point apparatus was used to obtain melting points and they were uncorrected. Elemental microanalyses were implemented at the microanalytical center at Al-Azhar University. IR spectra were recorded on Shimadzu IR 435 spectrometer KBr discs on 6 October at the Faculty of Pharmacy, University for Modern Sciences and Arts (MSA University), Giza, Egypt, and values are presented in cm^−1^. ^1^H and ^13^C NMR spectra were recorded using a Bruker (Bruker Corp., Billerica, MA, USA) spectrometer at (400 MHz ^1^H, 100 MHz ^13^C) at the Faculty of Pharmacy, Cairo University, Cairo, Egypt, and Faculty of Pharmacy, Ain Shams University, Cairo, Egypt, using Tetramethylsilane (TMS) as an internal-standard; chemical shifts were recorded in δ ppm and coupling constants (*J*) were reported in Hz. Thin-layer chromatography was carried out on silica gel TLC plates with a fluorescence indicator (F254). The spots were visualized using a UV lamp. The solvent system used for this assay was ethyl acetate: hexane (3:7). 

Compound **1** was prepared as described in the literature [28].

#### 3.1.2. Synthesis of 2-Chloro-N-(5-(4-Chlorophenyl)-1,3,4-Thiadiazol-2-yl) Acetamide (2) [29]

Compound **1** (2.11 g, 10 mmol) and anhydrous sodium acetate (0.80 g, 10 mmol) were added to dry acetone (30 mL), followed by the addition of chloroacetyl chloride (1.12 g, 10 mmol). The reaction mixture was stirred for 1 h in the cold and then poured into ice-cold water (100 mL); the formed precipitate was then filtered, washed with water, dried, and crystallized from ethanol.

The white crystals (2.50 g, 87%) decomposed at 250 °C and had an IR (KBr, ν_max_ cm^−1^): 3168 (NH), 3040 (CH aromatic), 2947 (CH aliphatic), 1706 (C = O), 1563 (C = C); ^1^H NMR (DMSO- *d*_6_, 400 MHz) δ: 4.49 (s, 2H, CH_2_), 7.60 (d, 2H, *J* = 8.16 Hz, ArH), 7.98 (d, 2H, *J* = 8.10 Hz, ArH), and 13.10 (s, 1H, NH, and D_2_O exchangeable).

#### 3.1.3. Synthesis of 1-(2-((5-(4-Chlorophenyl)-1,3,4-Thiadiazol-2-yl)Amino)-2-Oxoethyl)Pyridinium Chloride (3)

Compound **2** (0.29 g, 1 mmol) was refluxed in dry pyridine (10 mL) for 3 h. The reaction mixture was cooled and poured into dil. HCl. The formed precipitate was filtered, dried, and crystallized from ethanol.

The resulting white crystals (0.30 g, 81%), had a m.p. 279–281 °C; IR (KBr, ν_max_ cm^−1^): 3420 (NH), 3055–3020 (CH aromatic), 2875 (CH aliphatic), 1697 (C = O), 1638 (C = N), 1549 (C = C); ^1^H NMR (DMSO- *d*_6_, 400 MHz) δ: 6.00 (s, 2H, CH_2_), 7.58 (d, 2H, *J* = 9.20 Hz, ArH), 7.93 (d, 2H, *J* = 9.20 Hz, ArH), 8.27 (t, 2H, *J* = 8.80 Hz, ArH), 8.75 (t, 1H, *J* = 7.60 Hz, ArH), 9.19 (d, *J* = 5.20 Hz, 2H, ArH), and 13.76 (s, 1H, NH, and D_2_O exchangeable). ^13^C NMR (DMSO-*d*_6_, 100 MHz) δ: 62.32, 103.92, 109.05, 128.14, 129.14, 129.20, 129.97, 135.91, 147.05, 147.16, and 165.00. Anal. Calcd. for C_15_H_12_ClN ^+^ _4_OSCl^-^ (367.25): C, 49.01; H, 3.27; N, 15.25; found: C, 49.28; H, 3.43; and N, 15.53.

#### 3.1.4. General Procedures for the Synthesis of N-(5-(4-Chlorophenyl)-1,3,4-Thiadiazol-2-yl)-2-(Disubstitutedamino)Acetamide Derivatives (**4a–i**)

To a solution of **2** (0.29 g, 1 mmol) in dry benzene (30 mL), the appropriate secondary amine (1 mmol) was added, followed by TEA (0.2 mL). This mixture was heated under reflux for 16–20 h; the progress of the reaction was monitored by TLC. The formed precipitate was collected through filtration while hot, then dried and crystallized from ethanol. 

##### *N*-(5-(4-Chlorophenyl)-1,3,4-Thiadiazol-2-yl)-2-(4-Methylpiperazin-1-yl)Acetamide (**4a**) 

The white crystals (0.26 g, 74%) had a reaction time of 18 h, m.p. 168–170 °C; IR (KBr, ν_max_ cm^−1^): 3240 (NH), 3030–3019 (CH aromatic), 2900–2822 (CH aliphatic), 1705 (C = O), 1640 (C = N), 1593 (C = C); ^1^H NMR (DMSO- *d*_6_, 400 MHz) δ: 2.73 (s, 3H, CH_3_), 2.90 (br s, 4H, piperazine Hs), 3.41 (br s, 4H, piperazine Hs), 3.53 (s, 2H, CH_2_), 7.59 (d, 2H, *J* = 8.20 Hz, ArH), 7.96 (d, 2H, *J* = 8.24 Hz, ArH), 11.00, and 12.67 (2s, 1H, NH/OH, and D_2_O exchangeable). ^13^C NMR (DMSO-*d*_6_, 100 MHz) δ: 42.58, 49.46, 52.85, 59.22, 129.09, 129.46, 129.90, 135.67, 158.72, 161.26, and 169.07. Anal. Calcd. for C_15_H_18_ClN_5_OS (351.85): C, 51.20; H, 5.16; N, 19.90; found: C, 51.42; H, 5.37; and N, 19.73.

##### *N*-(5-(4-Chlorophenyl)-1,3,4-Thiadiazol-2-yl)-2-(4-Ethylpiperazin-1-yl)Acetamide (**4b**)

The buff crystals (0.27 g, 73%) had a reaction time of 18 h, m.p. 163–165 °C; IR (KBr, ν_max_ cm^−1^): 3276 (NH), 3035–3020 (CH aromatic), 2966–2880 (CH aliphatic), 1697 (C = O), 1630 (C = N), 1557 (C = C); ^1^H NMR (DMSO- *d*_6_, 400 MHz) δ: 1.00 (t, 3H, *J* = 7.20 Hz, CH_2_CH_3_), 2.41 (q, 2H, *J* = 7.20 Hz, CH_2_CH_3_), 2.47 (br s, 4H, piperazine Hs),2.58 (br s, 4H, piperazine Hs), 3.38 (s, 2H, CH_2_), 7.58 (d, 2H, *J* = 8.80 Hz, ArH), 7.95 (d, 2H, *J* = 8.80 Hz, ArH), and 10.50 (s, 1H, NH, and D_2_O exchangeable). ^13^C NMR (DMSO-*d*_6_, 100 MHz) δ: 12.13, 51.95, 52.46, 52.66, 60.42, 129.05, 129.52, 129.87, 135.61, 158.95, 161.16, and 169.33 Anal. Calcd. for C_16_H_20_ClN_5_OS (365.88): C, 52.52; H, 5.51; N, 19.14; found: C, 52.71; H, 5.68; and N, 19.42.

##### *N*-(5-(4-Chlorophenyl)-1,3,4-Thiadiazol-2-yl)-2-(4-Phenylpiperazin-1-yl)Acetamide (**4c**)

The buff crystals (0.26 g, 62%) had a reaction time of 20 h, m.p. 214–216 °C; IR (KBr, ν_max_ cm^−1^): 3245 (NH), 3020–3050 (CH aromatic), 2990–2825 (CH aliphatic), 1699 (C = O), 1630 (C = N), 1597 (C = C); ^1^H NMR (DMSO- *d*_6_, 400 MHz) δ: 2.70 (t, 4H, *J* = 4.80 Hz, piperazine Hs), 3.18 (t, 4H, *J* = 4.56 Hz, piperazine Hs), 3.46 (s, 2H, CH_2_), 6.78 (t, 1H, *J* = 7.28 Hz, ArH), 6.93 (d, 2H, *J* = 8.0 Hz, ArH), 7.21 (t, 2H, *J* = 7.36 Hz, ArH), 7.59 (d, 2H, *J* = 8.8 Hz, ArH), 7.97 (d, 2H, *J* = 8.8 Hz, ArH), and 12.41 (s, 1H, NH, and D_2_O exchangeable). ^13^C NMR (DMSO-*d*_6_, 100 MHz) δ: 48.62, 52.88, 60.42, 115.93, 116.50, 119.32, 128.79, 129.09, 129.40, 129.50, 129.90, 135.65, 151.44, 158.82, 161.25, and 169.28. Anal. Calcd. for C_20_H_20_ClN_5_OS (413.92): C, 58.03; H, 4.87; N, 16.92; found: C, 58.24; H, 5.01; and N, 17.14.

##### *N*-(5-(4-Chlorophenyl)-1,3,4-Thiadiazol-2-yl)-2-(4-(4-Tolyl)Piperazin-1-yl)Acetamide (**4d**) 

The white crystals (0.26 g, 61%) had a reaction time of 18 h, m.p. 222–224 °C; IR (KBr, ν_max_ cm^−1^): 3273 (NH), 3035 (CH aromatic), 2934–2825 (CH aliphatic), 1708 (C = O), 1631 (C = N), 1586 (C = C); ^1^H NMR (DMSO- *d*_6_, 400 MHz) δ: 1.25 (s, 3H, CH_3_), 2.88 (br s, 4H, piperazine Hs), 3.07 (br s, 4H, piperazine Hs), 3.52 (s, 2H, CH_2_), 7.49 (d, 2H, *J* = 7.60 Hz, ArH), 7.59 (d, 2H, *J* = 7.60 Hz, ArH), 7.76 (d, 2H, *J* = 9.60 Hz, ArH), 7.96 (d, 2H, *J* = 9.60 Hz, ArH), 10.61, and 12.51 (2s, 1H, NH/OH, and D_2_O exchangeable). ^13^C NMR (DMSO-*d*_6_, 100 MHz) δ: 9.37, 49.55, 51.09, 59.28, 128.37, 129.09, 129.47, 129.63, 129.90, 130.34, 134.41, 135.69, 158.74, 161.28, and 169.31. Anal. Calcd. for C_21_H_22_ClN_5_OS (427.95): C, 58.94; H, 5.18; N, 16.37; found: C, 59.11; H, 5.34; and N, 16.59.

##### *N*-(5-(4-Chlorophenyl)-1,3,4-Thiadiazol-2-yl)-2-(4-(2-Ethoxyphenyl)Piperazin-1-yl)Acetamide (**4e**)

The pale brown crystals (0.39 g, 85%) had a reaction time of 19 h, m.p. 200–202 °C; IR (KBr, ν_max_ cm^−1^): 3260 (NH), 3090–3040 (CH aromatic), 2910–2894 (CH aliphatic), 1702 (C = O), 1600 (C = N), 1573 (C = C); ^1^H NMR (DMSO- *d*_6_, 400 MHz) δ:1.19 (t, 3H, *J* = 7.20 Hz, OCH_2_CH_3_), 2.69 (br s, 4H, piperazine Hs), 3.05–3.10 (q, 2H, *J* = 7.20 Hz, CH_2_, OCH_2_CH_3_), 3.22 (br s, 4H, piperazine Hs), 3.46 (s, 2H, CH_2_), 6.77 (d, 1H, *J* = 9.60 Hz, ArH), 6.89–6.94 (m, 2H, ArH), 7.21 (t, 1H, *J* = 8.40 ArH), 7.59 (d, 2H, *J* = 8.40 Hz, ArH), 7.97 (d, 2H, *J* = 8.40 Hz, ArH), and 12.70 (s, 1H, NH, and D_2_O exchangeable). ^13^C NMR (DMSO-*d*_6_, 100 MHz) δ: 9.03, 46.15, 48.03, 52.64, 60.29, 103.95, 114.18, 115.04, 128.79, 129.10, 129.91, 130.91, 131.77, 134.28, 135.68, 140.49, 150.93, and 169.24. Anal. Calcd. for C_22_H_24_ClN_5_O_2_S (457.98): C, 57.70; H, 5.28; N, 15.29; found: C, 57.91; H, 5.47; and N, 15.48.

##### *N*-(5-(4-Chlorophenyl)-1,3,4-Thiadiazol-2-yl)-2-(4-(4-Ethoxyphenyl)Piperazin-1-yl)Acetamide (**4f**) 

The buff crystals (0.41 g, 89%) had a reaction time of 19 h, m.p. 225–227 °C; IR (KBr, ν_max_ cm^−1^): 3280 (NH), 3030 (CH aromatic), 2927–2823 (CH aliphatic), 1700 (C = O), 1625 (C = N), 1594 (C = C); ^1^H NMR (DMSO- *d*_6_, 400 MHz) δ: 1.20 (t, 3H, *J* = 7.20 Hz, OCH_2_CH_3_), 2.69 (br s, 4H, piperazine Hs), 3.03–3.08 (q, 2H, *J* = 7.20 Hz, OCH_2_CH_3_), 3.17 (br s, 4H, piperazine Hs), 3.46 (s, 2H, CH_2_), 6.92 (d, 2H, *J* = 8.40 Hz, ArH), 7.20 (d, 2H, *J* = 8.40 Hz, ArH), 7.57 (d, 2H, *J* = 8.00 Hz, ArH), 7.94 (d, 2H, *J* = 8.00 Hz, ArH), and 12.35 (s, 1H, NH, and D_2_O exchangeable). ^13^C NMR (DMSO-*d*_6_, 100 MHz) δ: 8.93, 45.92, 48.40, 52.68, 60.26, 117.36, 122.82, 128.36, 129.07, 129.49, 129.88, 135.65, 150.21, 158.77, 161.25, and 169.18. Anal. Calcd. for C_22_H_25_ClN_5_O_2_S (457.98): C, 57.70; H, 5.28; N, 15.29; found: C, 57.85; H, 5.17; and N, 15.03.

##### *N*-(5-(4-Chlorophenyl)-1,3,4-Thiadiazol-2-yl)-2-(4-(4-Fluorophenyl)Piperazin-1-yl)Acetamide (**4g**) 

The pale brown crystals (0.31 g, 72%) had a reaction time of 20 h, m.p. 203–205 °C; IR (KBr, ν_max_ cm^−1^): 3250 (NH), 3040 (CH aromatic), 2915–2816 (CH aliphatic), 1698 (C = O), 1648 (C = N), 1580 (C = C); ^1^H NMR (DMSO- *d*_6_, 400 MHz) δ: 2.72 (br s, 4H, piperazine Hs), 3.12 (br s, 4H, piperazine Hs), 3.48 (s, 2H, CH_2_), 6.95–7.06 (m, 4H, ArH), 7.58 (d, 2H, *J* = 9.60 Hz, ArH), 7.96 (d, 2H, *J* = 9.60 Hz, ArH), and 12.60 (s, 1H, NH, and D_2_O exchangeable). ^13^C NMR (DMSO-*d*_6_, 100 MHz) δ: 49.34, 52.85, 60.28, 115.61, 115.83, 117.65, 118.60, 128.78, 129.47, 135.67, 148.32, 158.79, 161.29, and 169.17. Anal. Calcd. for C_20_H_19_ClFN_5_OS (431.91): C, 55.62; H, 4.43; N, 16.22; found: C, 55.49; H, 4.68; and N, 16.49.

##### *N*-(5-(4-Chlorophenyl)-1,3,4-Thiadiazol-2-yl)-2-(4-(Furan-2-Carbonyl)Piperazin-1-yl)Acetamide (**4h**) 

The pale yellow crystals (0.34 g, 78%) had a reaction time of 20 h, m.p. 180–182 °C; IR (KBr, ν_max_ cm^−1^): 3210 (NH), 3080 (CH aromatic), 2938–2870 (CH aliphatic), 1703, 1620 (C = O), 1611 (C = N), 1590 (C = C); ^1^H NMR (DMSO- *d*_6_, 400 MHz) δ: 2.51–2.63 (m, 4H, piperazine Hs), 3.05–3.16 (m, 4H, piperazine Hs), 3.71 (s, 2H, CH_2_), 6.62 (d, 1H, *J*=1.20 Hz, ArH), 6.99 (t, 1H, *J*=3.30 Hz, ArH), 7.58 (d, 2H, *J* = 8.00 Hz, ArH), 7.83 (d, 1H, *J* = 2.60, ArH), 7.95 (d, 2H, *J* = 8.00 Hz, ArH), and 10.32 (s, 1H, NH, and D_2_O exchangeable). ^13^C NMR (DMSO-*d*_6_, 100 MHz) δ: 45.99, 52.80, 60.04, 111.78, 116.08, 129.08, 129.43, 129.90, 135.68, 145.16, 147.34, 158.73, 158.81, 161.32, and 169.26. Anal. Calcd. For C_19_H_22_ClN_5_O_3_S (435.93): C, 52.35; H, 5.09; N, 16.07; found: C, 52.52; H, 5.17; and N, 16.21.

##### 2-(4-Benzylpiperidin-1-yl)-*N*-(5-(4-Chlorophenyl)-1,3,4-Thiadiazol-2-yl)Acetamide (**4i**)

The white crystals (0.31 g, 73%) had a reaction time of 16 h, m.p. 162–164 °C; IR (KBr, ν_max_ cm^−1^): 3328 (NH), 3050–3020 (CH aromatic), 2985–2929 (CH aliphatic), 1680 (C = O), 1623 (C = N), 1593 (C = C); ^1^H NMR (DMSO- *d*_6_, 400 MHz) δ: 1.20–3.07 (m, 9H, piperidine Hs), 1.53 (d, 2H, *J* = 12.00 Hz, CH_2_), 3.39 (s, 2H, CH_2_), 7.17 (t, 2H, *J* = 7.16 Hz, ArH), 7.27 (t, 1H, *J* = 7.12, Hz, ArH), 7.52 (d, 2H, *J* = 7.28, ArH), 7.76 (d, 2H, *J* = 8.2 Hz, ArH), 7.95 (d, 2H, *J* = 8.2 Hz, ArH), and 10.49 (s, 1H, NH, and D_2_O exchangeable). ^13^C NMR (DMSO-*d*_6_, 100 MHz) δ: 31.72, 35.45, 37.10, 42.67, 45.75, 53.48, 60.76, 126.23, 128.36, 128.60, 129.01, 129.45, 129.86, 130.33, 134.39, 140.69, 155.54, 159.47, 160.92, and 169.39. Anal. Calcd. for C_22_H_23_ClN_4_OS (426.96): C, 61.89; H, 5.43; N, 13.12; found: C, 61.73; H, 5.61; and N, 13.40.

#### 3.1.5. General Procedures for the Synthesis of 4-((5-(4-Chlorophenyl)-1,3,4-Thiadiazol-2-yl)Imino)-*N*-(4-Substitutedphenyl)-4,5-Dihydrothiazol-2-Amine (**5a–e**)

Compound **2** (0.57 g, 2 mmol) was dissolved in absolute ethanol (25 mL), then anhydrous sodium acetate (0.16 g, 2 mmol) was added, followed by the addition of the appropriate thiourea derivative (2 mmol). This mixture was heated under reflux for 4–5 h and the reaction progress was monitored using TLC. The reaction mixture was poured into cold water (50 mL), and the formed precipitate was filtered, washed with water, dried, and crystallized from ethyl acetate.

##### 4-((5-(4-Chlorophenyl)-1,3,4-Thiadiazol-2-yl)Imino)-*N*-Phenyl-4,5-Dihydrothiazol-2-Amine (**5a**)

The yellowish white crystals (0.41 g, 54%) had a m.p. 226–228 °C; IR (KBr, ν_max_ cm^−1^): 3274 (NH), 3090 (CH aromatic), 1680, 1636 (C = N), 1569 (C = C); ^1^H NMR (DMSO- *d*_6_, 400 MHz) δ: 2.23 (s, 2H, CH_2_, thiazole Hs), 7.47–7.54 (m, 3H, ArH), 7.58 (d, 2H, *J* = 8.40 Hz, ArH), 7.76 (d, 2H, *J* = 8.00 Hz, ArH), 7.96 (d, 2H, *J* = 8.00 Hz, ArH), and 12.63 (s, 1H, NH, and D_2_O exchangeable). ^13^C NMR (DMSO-*d*_6_, 100 MHz) δ: 22.90, 128.38, 129.05, 129.56, 129.63, 129.87, 130.32, 134.43, 149.29, 150.65, 155.12, 155.60, 159.13, 161.02, and 169.31. Anal. Calcd. for C_17_H_12_ClN_5_S_2_ (385.89): C, 52.91; H, 3.13; N, 18.15; found: C, 53.07; H, 3.28; and N, 18.34.

##### 4-((5-(4-Chlorophenyl)-1,3,4-Thiadiazol-2-yl)Imino)-*N*-(4-Tolyl)-4,5-Dihydrothiazol-2-Amine (**5b**) 

The pale brown crystals (0.40g, 50%) had a m.p. 230–232 °C; IR (KBr, ν_max_ cm^−1^): 3280 (NH), 3165 (CH aromatic), 2924 (CH aliphatic), 1692, 1638 (C = N), 1559 (C = C); ^1^H NMR (DMSO- *d*_6_, 400 MHz) δ: 2.16 (s, 2H, CH_2_, thiazole Hs), 2.25 (s, 3H, CH_3_), 7.07 (d, 2H, *J* = 7.20 Hz, ArH), 7.39 (d, 2H, *J* = 8.40 Hz, ArH), 7.53 (d, 2H, *J* = 8.40 Hz, ArH), 7.90 (d, 2H, *J* = 7.20 Hz, ArH), and 10.60 (s, 1H, NH, and D_2_O exchangeable). ^13^C NMR (DMSO-*d*_6_, 100 MHz) δ: 20.93, 23.91, 123.35, 128.71, 129.21, 129.62, 130.32, 133.37, 134.93, 137.79, 159.61, 162.14, 171.11, and 181.70. Anal. Calcd. for C_18_H_14_ClN_5_S_2_ (399.92): C, 54.06; H, 3.53; N, 17.51; found: C, 54.23; H, 3.70; and N, 17.72.

##### 4-((5-(4-Chlorophenyl)-1,3,4-Thiadiazol-2-yl)Imino)-*N*-(4-Ethylphenyl)-4,5-Dihydrothiazol-2-Amine (**5c**)

The brown crystals (0.43 g, 52%) had a m.p. 232–234 °C; IR (KBr, ν_max_ cm^−1^): 3270 (NH), 3157 (CH aromatic), 2959 (CH aliphatic), 1693, 1620 (C = N), 1545 (C = C); ^1^H NMR (DMSO-*d*_6_, 400 MHz) δ: 1.17 (t, 3H, *J* = 7.60 Hz, CH_2_CH_3_), 2.22 (s, 2H, CH_2_, thiazole Hs), 2.54 (q, 2H, *J* = 7.60 Hz, CH_2_CH_3_), 7.15 (d, 2H, *J* = 8.00 Hz, ArH), 7.26 (d, 2H, *J* = 8.00 Hz, ArH), 7.58 (d, 2H, *J* = 8.40 Hz, ArH), 7.95 (d, 2H, *J* = 8.40 Hz, ArH), and 12.60 (s, 1H, NH, and D_2_O exchangeable). ^13^C NMR (DMSO-*d*_6_, 100 MHz) δ: 16.05, 22.87, 28.09, 123.84, 128.36, 128.43, 129.00, 129.52, 129.61, 129.83, 135.57, 137.10, 159.10, 161.03, and 169.23. Anal. Calcd. for C_19_H_16_ClN_5_S_2_ (413.94): C, 55.13; H, 3.90; N, 16.92; found: C, 55.28; H, 3.76; and N, 17.14.

##### 4-((4-((5-(4-Chlorophenyl)-1,3,4-Thiadiazol-2-yl)Imino)-4,5-Dihydrothiazol-2-yl)Amino)Phenol (**5d**)

The grey crystals (0.41 g, 51%) had a m.p. 220–222 °C; IR (KBr, ν_max_ cm^−1^): 3300–2970 (OH broadband), 1659, 1602, 1572 (C = N), ^1^H NMR (DMSO-*d*_6_, 400 MHz) δ: 3.89 (s, 2H, CH_2_, thiazole Hs), 6.77 (d, 2H, *J* = 8.40 Hz, ArH), 6.91 (d, 2H, *J* = 8.40 Hz, ArH), 7.47 (d, 2H, *J* = 9.60 Hz, ArH), 7.76 (d, 2H, *J* = 9.60 Hz, ArH), and 10.26 (br s, 2H, OH, and NH, D_2_O exchangeable). ^13^C NMR (DMSO-*d*_6_, 100 MHz) δ: 36.90, 115.75, 116.26, 122.50, 124.70, 128.37, 129.63, 130.31, 134.44, 155.03, 169.33, 177.65, and 188.48. Anal. Calcd. for C_17_H_12_ClN_5_OS_2_ (401.89): C, 50.81; H, 3.01; N, 17.43; found: C, 51.07; H, 3.23; and N, 17.60.

##### *N*-(4-Bromophenyl)-4-((5-(4-Chlorophenyl)-1,3,4-Thiadiazol-2-yl)Imino)-4,5-Dihydrothiazol-2-Amine (**5e**)

The pale brown crystals (0.52 g, 56%) had a m.p. 208–210 °C; IR (KBr, ν_max_ cm^−1^): 3274 (NH), 3091 (CH aromatic), 1674, 1635 (C = N), 1563 (C = C), ^1^H NMR (DMSO- *d*_6_, 400 MHz) δ: 4.01 (s, 2H, CH_2_, thiazole Hs), 7.23 (d, 2H, *J* = 8.40 Hz, ArH), 7.47 (d, 2H, *J* = 11.60 Hz, ArH), 7.52 (d, 2H, *J* = 11.60 Hz, ArH), 7.76 (d, 2H, *J* = 8.40 Hz, ArH), and 10.93 (s, 1H, NH, and D_2_O exchangeable). ^13^C NMR (DMSO-*d*_6_, 100 MHz) δ: 35.00, 116.28, 117.07, 125.37, 125.74, 128.37, 129.62, 130.26, 131.81, 132.60, 134.48, 155.71, and 169.33. Anal. Calcd. for C_17_H_11_BrClN_5_S_2_ (464.78): C, 43.93; H, 2.39; N, 15.07; found: C, 44.17; H, 2.60; and N, 14.96.

### 3.2. In Vitro Cytotoxicity Screening

#### 3.2.1. Cell Culture

Human hepatocellular carcinoma (HepG2) cells and breast adenocarcinoma (MCF7) cells were purchased from the American Type Culture Collection (ATCC, USA). Under 37 °C with 5% CO2 in a humid incubator, cells were propagated using RPMI supplemented with 10% fetal bovine serum (FBS) and 1% *v*/*v* penicillin-streptomycin.

#### 3.2.2. Cytotoxicity Assay

According to Tim Mosmann’s approach [30], an MTT assay was performed to assess the antiproliferative properties of the compounds. In short, HepG2 or MCF7 cells (0.5 × 105) were grown in flat-bottom 96-well microplates in 180 μL/well RPMI media supplemented with 10% fetal bovine serum, 2μmol/mL L-glutamine, 250 ng/mL fungizone, and 100 units/mL penicillin-streptomycin solutions at 37 °C in a CO_2_ incubator. Following incubation, 180 μL/well of the new serum-free medium was added to each well after the old media had been withdrawn. After that, cells were exposed to 20 μL of various concentrations of the compounds (50–0.75 μg/mL) and were incubated for 48 h at 37 °C in a humidified 5% CO_2_ environment. The MTT solution (20 µL/well) was added and incubated for a further 4 h. The plate was agitated at room temperature while acidified isopropanol (160 μL/well) was added to dissolve the formazan crystals. The absorbance at 570 nm was then determined photometrically using the microplate ELISA reader (FLUOstar Omega, BMG, Labtech, Germany). Graph-Pad PRISM version 6 was used to determine the IC_50_ for each compound.

### 3.3. Acridine Orange/Ethidium Bromide Fluorescent Stain

Fluorescence labeling with acridine orange/ethidium bromide (AO/EB) was used to determine the ratio between apoptosis and necrosis to evaluate the mode of cell death. HepG2 and MCF-7 cells were seeded in six-well plates with DMEM media supplemented with 10% FBS and allowed to settle overnight at 37 °C with 5% CO2 in a humidified incubator. HepG2 cells were treated with **4e** and MCF7 cells were treated with **4i** using IC_50_ concentrations and incubated for 48 h. 5-FU was used as a positive standard for both cell lines. After incubation, cells were collected by trypsinization and stained using a dye solution of AO and EB in PBS (1:1) for 20 min. The cells were examined using fluorescence microscopy [31]. Each sample had at least 500 counted cells, and the percentage of apoptotic or necrotic cells was determined by multiplying the number of apoptotic or necrotic cells by the total number of cells counted by 100. The experiment was performed three times in triplicate.

### 3.4. Apoptosis Analysis by Flowcytometry

The apoptotic activity of compounds **4e** and **4i** was measured using the annexin V fluorescein isothiocyanate (FITC)/propidium iodide (PI) apoptosis detection kit (BioVision, USA, Catalog #: K101-25) following the manufacturer’s instructions. Briefly, in a tissue culture flask, HepG2 or MCF-7 cells were treated with **4e** or **4i** compounds, respectively, using IC_50_ concentration for each compound for 48 h. Cells were collected by trypsinization, washed twice using PBS and then 1 × 10^5^ cells were resuspended in PBS and stained for 15 min at room temperature in the dark with 5 µL Annexin V-FITC and 5 µL PI in 1x binding buffer. Annexin V-FITC binding was analyzed by a BD FACS Calibur flow cytometry (Ex = 488 nm; Em = 530 nm) using a FITC signal detector (FL1) and PI staining by the phycoerythrin emission signal detector (FL2).

### 3.5. Cell Cycle Analysis

To evaluate cell cycle distribution, **4e** and **4i** compounds were applied to HepG2 and MCF-7 cells, respectively, for 48 h at their IC_50_ concentrations. Immediately after incubation, trypsinization was used to collect the cells, which were then washed twice with ice-cold PBS before being fixed in 70% (*v*/*v*) ethanol at 20 °C, re-suspended with 0.1 mg/mL RNase, stained with 40 mg/mL propidium iodide (PI), and incubated for 1 h. The DNA contents of the cells were then analyzed by flow cytometry using an FL2 (λex/em 493/636 nm) signal detector (Abcam, UK, ab139418).

### 3.6. ELISA Assays

The protein levels of Bax, Bcl2, caspase 9, and VGEF were estimated using an ELISA assay. Briefly, T-25, HepG2, and MCF-7 cells in tissue culture flasks were treated after confluency with **4e** and **4i** compounds respectively, or with 5-FU as a positive control using IC_50_ for 48 h. Cells from each group were scraped off and washed with PBS and then cell pellets were collected from each flask. Following three repeated cycles of freezing and thawing in ice-cold lysis buffer supplemented with a protease inhibitor cocktail, the cell lysate was prepared and then centrifuged at 14,000× *g* for 15 min at 4 °C. The supernatants were then used for ELISA assays in accordance with the manufacturer’s instructions for the ELISA kits (Sunlong Biotech, China), and absorbance and calculations were carried out using an ELISA reader (FLUOstar Omega, BMG, Labtech, Germany).

### 3.7. Radiosynthesis and In Vivo Biodistribution of Radioiodinated-4i

#### 3.7.1. Radiosynthesis of Radioiodinated-**4i**

Radioiodine was introduced electrophilically in compound **4i** by using chloramine-T as an efficient oxidizing agent to convert the radioactive iodide form into a radioactive iodonium form [32,33,34,35]. The radiosynthesis yield was optimized through an intensive study of different variants:

pH: in the range of 5–9

Chloramine-T amount: in the range of 200–1200 µg

Compound **4i** amount: in the range of 10–100 µg

Reaction time: in the range of 5–00 min

Radioiodinated-**4a** compound formation was evaluated via ascending paper chromatography (P.C.) and TLC to calculate the highest radiosynthesis yield. One-way ANOVA was used as a statistical test to evaluate data differences (level of significance set at *p* < 0.05).

#### 3.7.2. Radioactive In Vivo Tracing Studies of the Radioiodinated-**4i** Compound in a Sarcoma-Bearing Mice Model

##### Sarcoma Induction in Mice Model

The Ehrlich cell line was used to induce sarcoma in the right lateral thigh muscle of the mice by intramuscularly injecting 200 mL of the Ehrlich cell line concentrated suspension; swelling appeared three days later [36].

#### Biodistribution Studies of Radioiodinated-**4i** in Sarcoma-Bearing Mice Model

The animal study regulation for performing all preclinical studies was approved by the Egyptian Atomic Energy Authority through the animal ethics committee and all its regulations were followed intensively. Sarcoma-bearing female mice (20–25 g) were collected in groups of five and fed with food and water. Volumes of 200 μL radioiodinated-**4i** compound that contained about 6 MBq were IV injected in female Swiss albino mice with a solid tumor in the thigh muscle. After anesthetizing all mice via chloroform, they were dissected at 0.25–3 h post-injection. All body organs and fluids were collected and weighed; then, their radioactivities were counted using a NaI (Tl) crystal gamma counter. Blood, bone, and muscles were evaluated in percentages of 7, 10, and 40% of the total body weight, respectively [37]. Percent-injected dose per organ (% ID/organ ± S.D.) at each time point for a population of five mice were reported. A one-way ANOVA test was used to evaluate data differences (*p* < 0.05).

## 4. Conclusions

A new series of 5-(4-chlorophenyl)-1,3,4-thiadiazole-based compounds featuring pyridinium (**3**), substituted piperazines (**4a–g**), benzyl piperidine (**4i**), and aryl aminothiazoles (**5a–e**) heterocycles were designed and synthesized as potential anticancer agents. Compounds **4e** and **4i** remarkably reduced the cell proliferation of the MCF-7 and HepG2 cell lines with IC_50_ values of 5.36 and 2.32 µg/mL for MCF-7 cells and IC_50_ values of 3.13 and 6.51 µg/mL for the HepG2 cell line, respectively. The cytotoxicity of **4e** and **4i** towards the normal mammalian Vero cell line was also tested, and they were found to be selectively more toxic to cancer cell lines than normal cells. Additionally, cell cycle analysis showed that the most active compounds, **4e** and **4i,** were able to disrupt HepG2 and MCF-7 cell cycle phase distribution, inducing cellular accumulation in the S and G2/M phases, respectively. In addition, evaluation of the Bax/Bcl2 ratio and caspase 9 levels in HepG2 and MCF-7 before and after treatment with the most active compounds **4e** and **4i** demonstrated an increase in the Bax/Bcl2 ratio and in the concentration of apoptotic protein caspase 9, which are hallmarks for programmed cell death induction via apoptosis. Finally, radioiodinated-**4i** showed high selectivity toward sarcoma in muscle compared to normal muscle in a tumor-bearing mice model which proves the ability of compound **4i** to target tumor cells efficiently.

## Figures and Tables

**Table 1:** The cytotoxic activities of compounds **3**, **4a–i**, **5a–e,** and 5-FU on MCF-7 and HepG2 human cancer cell lines.

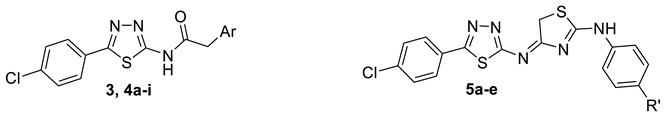			
**Compound**	**Ar/R’**	**IC_50_ (µg/mL)**	
		**MCF-7**	**HepG2**
**3**	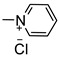	7.56 ± 1.20	13.00 ± 0.11
**4a**	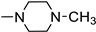	51.56 ± 0.48	26.30 ± 5.76
**4b**	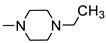	25.21 ± 1.30	3.55 ± 0.05
**4c**	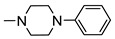	49.40 ± 3.50	27.94 ± 0.93
**4d**	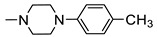	21.75 ± 0.43	44.87 ± 1.54
**4e**	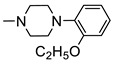	5.36 ± 0.36	3.13 ± 0.20
**4f**	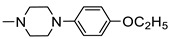	18.39 ± 0.85	38.10 ± 0.14
**4g**	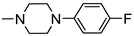	10.10 ± 1.20	13.87 ± 0.59
**4h**	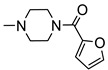	3.21 ± 0.30	8.35 ± 0.15
**4i**	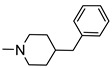	2.32 ± 0.21	6.51 ± 0.03
**5a**	H	24.79 ± 2.30	26.12 ± 2.02
**5b**	CH_3_	12.64 ± 0.47	8.81 ± 0.64
**5c**	CH_2_CH_3_	5.72 ± 0.17	11.25 ± 0.44
**5d**	OH	6.12 ± 0.51	14.12 ± 0.01
**5e**	Br	3.77 ± 0.13	25.77 ± 0.64
**5-FU**	_	6.80 ± 0.90	8.40 ± 1.20

IC_50_: concentration of the tested compound that inhibits 50% of cell growth. All data are presented as the means ± SD of three independent experiments.

**Table 2:** The cytotoxic activities of compounds **4e** and **4i** on MCF-7, HepG2, and Vero cell lines and the calculated selectivity indices.

**Compound**	**IC_50_ (µg/mL)**			**SI Values**	
	**MCF-7**	**HepG2**	**Vero**	**MCF-7**	**HepG2**
**4e**	5.36 ± 0.36	3.13 ± 0.20	154.30 ± 32.20	28.79	49.20
**4i**	2.32 ± 0.21	6.51 ± 0.03	85.29 ± 0.52	36.70	13.10

SI $=\frac{\mathrm{IC}50\;\mathrm{against}\;\mathrm{MCF}-7\;\mathrm{or}\;\mathrm{HepG}2\;\mathrm{cells}}{\mathrm{IC}50\;\mathrm{against}\;normal\;\mathrm{Vero}\;\mathrm{cells}\;}$.

## Data Availability

Data is contained within the article.
